# Medical Convention of Kentucky

**Published:** 1840-12

**Authors:** 


### MEDICAL CONVENTION OF KENTUCKY.

On the 22d of November, 1839, the physicians of North Eastern Kentucky held a meeting at Washington, and among other resolutions adopted the following:

"*Resolved*, That this Association respectfully urge upon the physicians of Kentucky, the expediency of forming district and county societies, for the promotion of medical science; and also, that a State Convention be held in Frankfort, on the second Monday in January, 1841, for the purpose of organizing a State Medical Society."

As the time for the proposed Convention is approaching, we beg leave to invite the attention of our Kentucky readers, not only to the meeting, but to a consideration of the objects which ought to receive attention. The gentlemen who have called it, had in view the "organizing of a State Medical Society;" but when they and their brethren assemble, it will be as competent for them to do any thing else, as that which has been named.

The formation of district or county societies and a State Society, may be attended with benefits to the profession; but we are obliged to confess, that our observations on the *modus operandi*, and effects of that organization, in a neighbouring State have not given us a very high opinion of its adaptation to the West. It was there kept up, as a kind of forced condition, for many years, during which no results were obtained that could, to a considerable degree, gratify any one who looked to more than mere formal meetings, thinly and reluctantly attended. We admit that this may have been less the fault of the system, than of the physicians among whom it was introduced; but it signifies little where the blame lies, if good fruits are not shed upon the community. A radical difficulty of this method is its complexity.

Another, exempt from this objection, is the voluntary meeting of any or all of the physicians, once a year, on the plan of the British Scientific Association, borrowed, we understand, from Germany. Such meetings might be held at Frankfort, or, successively, in the different large towns of the State, whereby all our brethren would be, progressively, included. A Convention of this kind would be generally attended, by a much larger number of the profession, than the meetings of a society composed of delegates, and its recommendations would, we are disposed to think, be more authoritative, while the simplicity of the whole operation, would be calculated to secure its perpetuity. We are, however, far from having formed a definitive opinion on the relative merits of the two methods, and have thrown out these hints for the purpose of exciting those who may design to visit Frankfort, to reflection on the subject.

That much might be done to arouse the profession of the State into greater activity, as well as to promote a feeling of brotherly kindness, and enkindle an *esprit du corps*, cannot be doubted. The question is as to the mode; and on this point, we trust that the respectable gentlemen who have proposed the meeting, for the second Monday of January, will come prepared with some well digested plan. D.

